# Long-term exposure to low concentrations of ambient benzene with incident thyroid dysfunction and the mediating role of accelerated biological aging

**DOI:** 10.3389/fpubh.2026.1964801

**Published:** 2026-09-17

**Authors:** Liliang Yu, Changlai Lyu, Meiqi Liu, Bin Wang, Daikun Zheng, Yunhao Liu

**Affiliations:** 1 Hubei Province Key Laboratory of Occupational Hazard Identification and Control, Department of Environmental Hygiene and Occupational Medicine, School of Public Health, Healthy Hubei Development and Social Progress Research Center of the Key Research Base of Humanities and Social Sciences in Hubei Province, Wuhan University of Science and Technology, Wuhan, Hubei, China; 2 Department of Preventive Medicine, School of Public Health and Management, Chongqing Three Gorges Medical College, Chongqing, China; 3 Department of Mathematics/Department of Computer Science and Engineering (CSE), The Hong Kong University of Science and Technology, Hong Kong, China; 4 School of Public Health, Tongji Medical College, Huazhong University of Science and Technology, Wuhan, Hubei, China

**Keywords:** accelerated biological aging, air pollutant, benzene, hyperthyroidism, hypothyroidism, thyroid dysfunction

## Abstract

**Background:**

The associations between ambient benzene exposure and thyroid dysfunction (hypothyroidism and hyperthyroidism), as well as the role of biological aging, remain largely unknown.

**Methods:**

We included a total of 385,130 participants from the UK Biobank, and Cox regressions were used to assess the association between benzene exposure and incident thyroid dysfunction. We employed restricted cubic splines to fit potential nonlinear exposure–response relationships. Furthermore, interactions and mediation analyses were conducted to examine the mediating role of biological aging.

**Results:**

Benzene exposure was dose-responsively associated with an increased risk of thyroid dysfunction, each 1 μg/m^3^ increase in benzene concentration was linked to hazard ratios (HRs) of 1.19 (95% CI: 1.16, 1.21) for hypothyroidism and 1.21 (95% CI: 1.18, 1.25) for hyperthyroidism. Participants with higher benzene exposure and older biological age showed a greater risk for hypothyroidism compared to those with lower exposure and younger biological age, with a relative excess risk (RERI) of 0.40 (95% CI: 0.13, 0.66). Furthermore, accelerated biological aging significantly mediated the association between benzene exposure and hyperthyroidism. Comparing the highest to the lowest quartile of benzene exposure, the proportion mediated (PM) (95% CI) was 3.07% (2.05, 4.08%).

**Conclusion:**

Long-term exposure to low concentrations benzene may increase the risk of incident thyroid dysfunction. Accelerated biological aging demonstrated a significant additive interaction with benzene exposure on the risk of hypothyroidism and partially mediated the positive association between benzene exposure and the risk of hyperthyroidism.

## 1 Introduction

Thyroid dysfunction refers to metabolic abnormalities caused by excessive or insufficient secretion of thyroid hormones, including primarily hyperthyroidism and hypothyroidism (1, 2). Normal thyroid function is essential for regulating both physiological and pathological processes, and alterations in thyroid hormone levels can significantly affect health (3, 4). Studies have shown that thyroid dysfunction is associated with various diseases, including hematologic disorders (5) and cardiovascular diseases (6–8). In recent years, the incidence of thyroid diseases has risen rapidly among adolescents and adults worldwide, with over 237,000 new cases of thyroid cancer reported globally in 2022 alone (9). According to the National Health and Nutrition Examination Survey (NHANES), the prevalence of thyroid dysfunction in the United States exceeds 8% (10). Various factors contribute to thyroid dysfunction, including unhealthy lifestyle habits, surgical interventions, and medication use (4, 11). Likewise, the impact of environmental pollutants on thyroid function has become a growing public health concern (12–14).

Benzene, a volatile organic compound and a common air pollutant (15), is recognized by the International Agency for Research on Cancer as a Group 1 carcinogen (16). Environmental benzene primarily originates from human activities, including vehicle exhaust, building and decorative materials, industrial emissions, and the use of cleaning agents (17, 18). Low concentrations of benzene and its metabolites have been widely detected in human biological samples (19, 20). Evidence indicates that exposure to environmental benzene is implicated in a heightened risk of various disorders, including hematopoietic dysfunction (21), respiratory disorders (22), and diabetes (23). The mechanisms potentially linking these associations, which encompass oxidative stress, chronic inflammation, and genetic factors, have been extensively investigated (24, 25). Furthermore, recent studies found long-term exposure to low levels of benzene has been linked to elevated mortality from a range of conditions, including cardiovascular and respiratory diseases (26). Despite substantial research on the health effects of benzene, few studies explored its association with thyroid diseases. To date, only one cross-sectional study has indicated altered thyroid hormone levels in gas station workers with occupational exposure to benzene-containing solvent mixtures (27). However, whether environmental exposure to low concentrations of benzene affects the risk of thyroid disease and the underlying mechanisms remains unknown.

In addition, aging is characterized by a progressive decline in an organism’s physiological and psychological adaptability to the environment, ultimately culminating in mortality. The rate of human aging varies considerably due to genetic heterogeneity and environmental influences (28, 29). In recent decades, thyroid dysfunction has seen a steady rise among older adults, making it a highly common condition within this demographic (30–32). In recent years, the assessment of biological age to determine an individual’s actual aging status has gained widespread acceptance (33, 34). Concurrently, studies have found that occupational benzene exposure is associated with accelerated biological aging (35), and that biological aging increases the risk of stroke related to benzene exposure (22). However, research evaluating the impact of biological aging on the relationship between benzene exposure and thyroid dysfunction remains limited.

Therefore, we aimed to prospectively investigate the association between long-term benzene exposure and the risk of thyroid dysfunction, and to examine further the interplay and joint influence of biological aging and benzene exposure on this risk.

## 2 Materials and methods

### 2.1 Study population

The study population was derived from the UK Biobank, a large-scale biomedical database that recruited approximately 500,000 participants aged 40–69 years across the United Kingdom between 2006 and 2010. All participants provided informed consent, and detailed information regarding the UK Biobank is available online.^1^ This cohort study received ethical approval from the North West Multicenter Research Ethics Committee and was conducted in accordance with the Declaration of Helsinki. Data from 502,292 participants were analyzed. After excluding 55,246 individuals lacking environmental benzene exposure data, 20,448 with self-reported thyroid cancer or non-malignant thyroid disease (based on medical history or hospital records), and 41,468 with incomplete covariate information, a total of 385,130 participants were included to assess the association between benzene exposure and thyroid dysfunction. Furthermore, after excluding 70,926 individuals lacking biological aging data, 314,204 participants were included to evaluate the potential role of accelerated biological aging in the relationship between benzene exposure and thyroid dysfunction (Supplementary Figure S1).

### 2.2 Exposure assessment

Average annual concentrations of benzene for the period 2006–2020 were derived from high-resolution, near-surface air pollution data provided by the United Kingdom Department for Environment, Food and Rural Affairs (DEFRA). National annual concentration maps at a 1 km × 1 km resolution were generated using an air dispersion model that integrates automatic monitoring data from background sites across DEFRA’s rural and urban networks, national emission inventories, and other relevant atmospheric factors (22). Individual exposure assessment was based on participants’ residential addresses from the UK Biobank. Each participant’s geocoded address was linked to the corresponding 1 km × 1 km grid cell of the annual benzene concentration maps. For participants who relocated during the study period, a time-weighted average exposure was calculated to account for varying residence durations at each address, thereby providing an integrated estimate of long-term personal benzene exposure.

### 2.3 Outcome and covariates

Incident thyroid dysfunction during follow-up was ascertained using International Classification of Diseases, 10th Revision (ICD-10) codes, based on the first recorded diagnosis in hospital inpatient records. This included hyperthyroidism (E050–E053, E055, and E058–E059) and hypothyroidism (E039).

The primary covariates examined in this study included age (years), sex (male and female), ethnicity (white people and others), Townsend deprivation index (TDI) (low, moderate, high), body mass index (BMI) (<30 kg/m^2^, ≥30 kg/m^2^), tobacco smoking (never, current or former smokers), alcohol consumption (never, current or former drinkers), physical activity (inactive, low, moderate, high), health diet (yes, no), and use of thyroid-related medications (yes, no), PM_2.5_. Here, a healthy diet was derived from seven dietary components based on a questionnaire survey (Supplementary Table S1). A score of 1 was assigned if the target for a given dietary component was met; otherwise, 0. The total score ranged from 0 to 7, and a diet with a total score ≥4 was defined as a healthy diet. Covariate selection was guided by a directed acyclic graph (DAG) constructed based on established risk factors for thyroid disease identified in the literature and prior epidemiological knowledge (Supplementary Figure S2).

### 2.4 Biological aging assessment

We employed the validated PhenoAge algorithm to calculate each participant’s biological age, as this metric reflects multisystem physiological decline. This algorithm was originally developed by applying elastic-net Gompertz regression to 42 clinical biomarkers to estimate mortality risk in the United States National Health and Nutrition Examination Survey (NHANES) III (36, 37). PhenoAge values were computed directly using the publicly available “BioAge” R package, which encodes the published algorithm and its mortality risk transformation (33). To isolate the component of accelerated biological aging independent of chronological age, we calculated accelerated biological aging as the residual from regressing the computed PhenoAge on chronological age. Consequently, a positive residual indicates an individual whose biological age is older than their chronological age (accelerated aging), while a negative residual indicates a biologically younger profile (decelerated aging).

### 2.5 Statistical analysis

Baseline characteristics of participants were described, with continuous variables presented as means and standard deviations, and categorical variables expressed as percentages. The associations between environmental benzene exposure (continuous and quartiles) and thyroid dysfunction were assessed using Cox proportional hazards models, estimating HRs and 95% CIs. Model 1 was adjusted for age and sex. Model 2 was further adjusted for additional covariates, including the TDI, BMI, smoking status, alcohol consumption, physical activity, and a healthy diet score. Model 3 was additionally adjusted for environmental PM_2.5_ exposure and thyroid-related medication use. A restricted cubic spline (RCS) containing three knots, with knot locations defined at the 10th, 50th, and 90th percentiles of the benzene exposure distribution, was constructed following the Akaike Information Criterion to assess the potential linear/non-linear dose–response relationship between benzene exposure and the risk of thyroid dysfunction. Furthermore, stratified analyses were performed according to age (<60 years, ≥60 years) and sex (female, male), ethnicity (white people and others), TDI (low, moderate, high), BMI (<30 kg/m^2^, ≥30 kg/m^2^), tobacco smoking (never, current or former smokers), alcohol consumption (never, current or former drinkers), physical activity (inactive, low, moderate, high), health diet (yes, no), and use of thyroid-related medications (yes, no), PM_2.5_ (low, high)_._

Multivariable-adjusted Cox proportional hazards models were used to assess the associations between biological aging and benzene exposure and the risk of thyroid dysfunction. Environmental benzene exposure was categorized into quartiles, and biological aging was dichotomized into biologically younger and biologically older. Using the combination of the lowest benzene quartile and biologically younger as the reference, their joint effects on thyroid dysfunction were examined. Additive interaction between benzene exposure and biological aging was evaluated using the relative excess risk due to interaction (RERI) and the attributable proportion due to interaction (AP), along with their 95% confidence intervals. Furthermore, in mediation analyses, we utilized the R package regmedint to investigate whether accelerated biological aging mediated the association between benzene concentration and the increased risk of thyroid dysfunction.

Multiple sensitivity analyses were conducted to verify the robustness of the primary findings. First, 41,468 participants with missing covariates were re-included in the analysis, and multiple imputation was performed to account for the expected bias due to missing data. Second, participants with less than 2 years of follow-up were excluded to reduce the potential influence of reverse causation. Third, participants with medication use were excluded (38). Fourth, inverse probability weighting was applied to adjust for covariates and minimize potential selection bias (39). Fifth, to address potential residual confounding from co-occurring air pollutants, an air pollution score was derived from PM_2.5_, PM_2.5–10_, NO_2_, and NO using principal component analysis; the first two principal components were weighted by their explained variances to construct the score, following previously established methods. This score was then used as an alternative adjustment in place of PM_2.5_ in a sensitivity analysis to assess the robustness of the benzene-thyroid association to co-pollutant adjustment. All statistical analyses were performed using R software, version 4.5.0. A two-sided *p*-value of less than 0.05 was considered statistically significant.

## 3 Results

### 3.1 Baseline characteristics

Among the 385,130 participants included, 7,675 (1.99%) cases of hyperthyroidism and 9,778 (2.54%) cases of hypothyroidism were identified during a median follow-up of 13.68 years (Table 1). The baseline characteristics of the included and excluded populations are presented in Supplementary Figure S1. In the overall study population, the mean (SD) age and PhenoAge were 56.55 (8.10) and 50.99 (9.97), respectively, while the mean (SD) PM_2.5_ concentration was 9.99 (1.05) μg/m^3^. Females accounted for 52.44% of the participants, 91.52% were White, 45.04% were smokers, and 95.69% reported alcohol consumption.

**Table 1:** Baseline characteristics of study participants.

Characteristics	Total participants (*N* = 385,130)	Participants with hyperthyroidism (*N* = 7,675)	Participants with hypothyroidism (*N* = 9,778)
Age (years), Mean (SD)	56.55 (8.10)	59.44 (7.81)	58.52 (7.63)
Sex (%)			
Female	201,979 (52.44%)	4,339 (56.53%)	6,942 (71.00%)
Male	183,151 (47.56%)	3,336 (43.47%)	2,836 (29.00%)
Ethnic group (%)			
Others	32,672 (8.48%)	1,057 (13.77%)	826 (8.45%)
White people	352,458 (91.52%)	6,618 (86.23%)	8,952 (91.55%)
TDI (%)			
Low TDI	204,223 (53.03%)	3,139 (40.90%)	4,829 (49.39%)
Medium TDI	121,424 (31.53%)	2,480 (32.31%)	3,211 (32.84%)
High TDI	59,483 (15.44%)	2,056 (26.79%)	1,738 (17.77%)
Physical activity (%)			
Inactive	5,922 (1.54%)	224 (2.92%)	212 (2.17%)
Low	83,825 (21.77%)	1,675 (21.82%)	2,077 (21.24%)
Moderate	202,323 (52.53%)	4,170 (54.33%)	5,354 (54.76%)
High	93,060 (24.16%)	1,606 (20.93%)	2,135 (21.83%)
BMI (kg/m^2^), Mean (SD)	27.38 (4.74)	28.72 (5.78)	28.33 (5.32)
Tobacco smoking (%)			
Never	211,668 (54.96%)	3,678 (47.92%)	5,093 (52.09%)
Current or former smokers	173,462 (45.04%)	3,997 (52.08%)	4,685 (47.91%)
Alcohol consumption (%)			
Never	16,609 (4.31%)	670 (8.73%)	679 (6.94%)
Current or former drinkers	368,521 (95.69%)	7,005 (91.27%)	9,099 (93.06%)
Healthy diet (%)			
No	57,559 (14.95%)	1,431 (18.64%)	1,372 (14.03%)
Yes	327,571 (85.05%)	6,244 (81.36%)	8,406 (85.97%)
Thyroid-related medications (%)	3,582 (0.93%)	128 (1.67%)	974 (9.96%)
PM_2.5_, Mean (SD)	9.99 (1.05)	10.22 (1.09)	10.05 (1.06)
PhenoAge (years)^a^, Mean (SD)	50.99 (9.97)	56.14 (10.10)	54.07 (9.66)
PhenoAge acceleration (years)^a^, Mean (SD)	−5.59 (5.31)	−3.32 (6.45)	−4.50 (5.83)

TDI, townsend deprivation index; PM_2.5_, particulate matter with a diameter <2.5 μm; ^a^The assessment of biological age included 314,204 participants.

### 3.2 Association of environmental benzene exposure with thyroid dysfunction

Table 2 demonstrates the association between benzene concentration and thyroid dysfunction. After multivariable adjustment, each 1 μg/m^3^ increase in benzene concentration was associated with an HR (95% CI) of 1.19 (1.16, 1.21) for hypothyroidism and 1.21 (1.18, 1.25) for hyperthyroidism. Compared with the lowest benzene concentration group, the highest concentration group had an HR (95% CI) of 1.58 (1.48, 1.68) for hypothyroidism and 1.60 (1.49, 1.72) for hyperthyroidism. Furthermore, we revealed a dose–response relationship between benzene concentration and thyroid dysfunction. After multivariable adjustment, benzene exposure showed a significant linear positive correlation with the incidence of hyperthyroidism (*p* for nonlinearity >0.05), whereas its association with hypothyroidism was nonlinear (*p* for nonlinearity < 0.001) (Figure 1).

**Table 2:** HR (95% CI) for incidence based on benzene concentration.

Model	Benzene (continuous)	Benzene (quartiles)				
	HR (95% CI) for 1 μg/m^3^ increase	Quartile 1	Quartile 2	Quartile 3	Quartile 4	*p* for trend
Hypothyroidism						
Model 1	1.19 (1.17, 1.22)	Ref.	1.32 (1.24, 1.40)	1.41 (1.33, 1.50)	1.67 (1.57, 1.77)	<0.001
Model 2	1.18 (1.16, 1.21)	Ref.	1.31 (1.23, 1.39)	1.38 (1.30, 1.47)	1.59 (1.49, 1.69)	<0.001
Model 3	1.19 (1.16, 1.21)	Ref.	1.30 (1.23, 1.38)	1.38 (1.30, 1.46)	1.58 (1.48, 1.68)	<0.001
Hyperthyroidism						
Model 1	1.39 (1.36, 1.42)	Ref.	1.29 (1.21, 1.39)	1.35 (1.26, 1.44)	2.26 (2.11, 2.41)	<0.001
Model 2	1.23 (1.20, 1.26)	Ref.	1.26 (1.17, 1.35)	1.23 (1.14, 1.32)	1.66 (1.54, 1.78)	<0.001
Model 3	1.21 (1.18, 1.25)	Ref.	1.24 (1.16, 1.33)	1.20 (1.12, 1.29)	1.60 (1.49, 1.72)	<0.001

Model 1: Age (continuous, years) and sex (female, male) were adjusted.

Model 2: Age (continuous, years), sex (male and female), ethnicity (white people and others), TDI (low, moderate, high), BMI (<30 kg/m^2^, ≥30 kg/m^2^), tobacco smoking (never, current or former smokers), alcohol consumption (never, current or former drinkers), physical activity (inactive, low, moderate, high), health diet (yes, no) were adjusted.

Model 3: Age (continuous, years), sex (male and female), ethnicity (white people and others), TDI (low, moderate, high), BMI (<30 kg/m^2^, ≥30 kg/m^2^), tobacco smoking (never, current or former smokers), alcohol consumption (never, current or former drinkers), physical activity (inactive, low, moderate, high), health diet (yes, no), and use of thyroid-related medications (yes, no), PM_2.5_ were adjusted.

HR, hazard ratio; CI, confidence interval; Ref., reference.

*p* for trend was tested by using ordered integer values.

**Figure 1:**
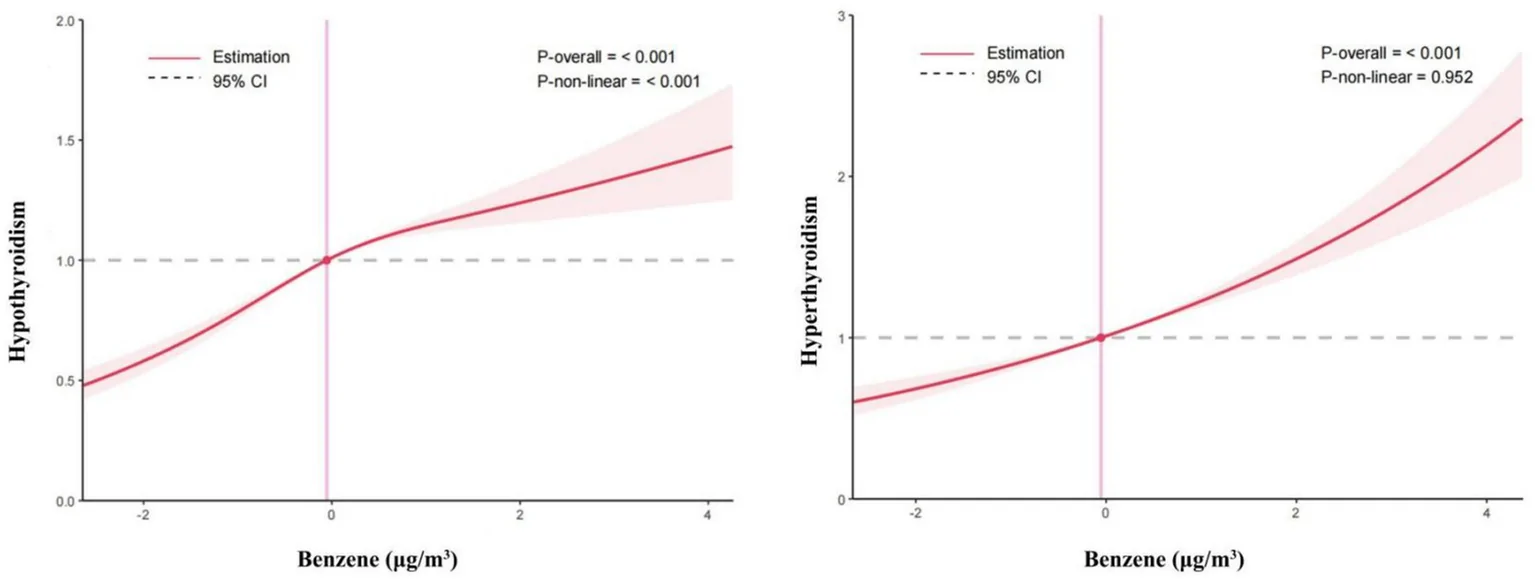
Exposure-response curves of environmental benzene exposure and thyroid dysfunction. Models were adjusted for age (continuous, years), sex (male and female), ethnicity (white people and others), TDI (low, moderate, high), BMI (<30 kg/m^2^, ≥30 kg/m^2^), tobacco smoking (never, current or former smokers), alcohol consumption (never, current or former drinkers), physical activity (inactive, low, moderate, high), health diet (yes, no), and use of thyroid-related medications (yes, no), PM_2.5_. BMI, body mass index; TDI, townsend deprivation index; PM_2.5_, particulate matter with a diameter of <2.5 μm.

Furthermore, stratified analyses by age, TDI, smoking status, and PM_2.5_ levels revealed that the association between long-term ambient benzene exposure and thyroid dysfunction risk varied across these subgroups (Supplementary Figure S3). Specifically, the risk of hyperthyroidism was higher in individuals younger than 60 years, while the risk of hypothyroidism was greater in those older than 60 years. Subjects with a higher TDI were more susceptible to thyroid dysfunction compared to those with a lower TDI. Non-smokers exhibited a higher risk of hyperthyroidism than smokers.

### 3.3 Association of accelerated biological aging with thyroid dysfunction

Accelerated biological aging significantly increased the risk of both hypothyroidism and hyperthyroidism (all *p* for trend <0.001) (Supplementary Table S2). After multivariable adjustment, compared with subjects in the lowest quartile of accelerated biological aging, those in the highest quartile had an HR (95% CI) of 1.49 (1.39, 1.59) for hypothyroidism and 1.93 (1.79, 2.09) for hyperthyroidism. Additionally, dose–response analysis showed that the relationship between accelerated biological aging and hypothyroidism exhibited a nonlinear increase (*p* for nonlinearity <0.05). In contrast, it showed a linear increase with hyperthyroidism (*p* for nonlinearity >0.05) (Supplementary Figure S4).

### 3.4 Role of accelerated biological aging in environmental benzene exposure and thyroid dysfunction

Dose–response analysis revealed an inverted U-shaped relationship between benzene exposure and accelerated biological aging, where biological aging accelerated with increasing benzene concentrations below 0.5 (Supplementary Figure S5). Given that both benzene exposure and accelerated biological aging are potential risk factors for thyroid dysfunction, we investigated their joint and interactive effects. We observed a significant additive interaction between environmental benzene exposures and accelerated biological aging on the risk of hypothyroidism (Figure 2, Supplementary Table S3). Specifically, compared with the lowest quartile of benzene concentration, the RERI (95% CI) for the third quartile and the highest quartile were 0.43 (0.18, 0.68) and 0.40 (0.13, 0.66), respectively, and the AP (95% CI) were 0.23 (0.11, 0.35) and 0.19 (0.07, 0.30), respectively. In addition, we found that the association between benzene concentration and increased risk of hyperthyroidism was partially mediated by accelerated biological aging (Table 3). Specifically, compared with the lowest quartile of benzene concentration, the PM (95% CI) for the second, third, and highest quartiles were 2.35 (0.88, 3.83), 5.00 (2.10, 7.80), and 3.07 (2.05, 4.08), respectively. Although statistically significant, the mediating role of accelerated biological aging in the association between benzene exposure and hypothyroidism was minimal.

**Figure 2:**
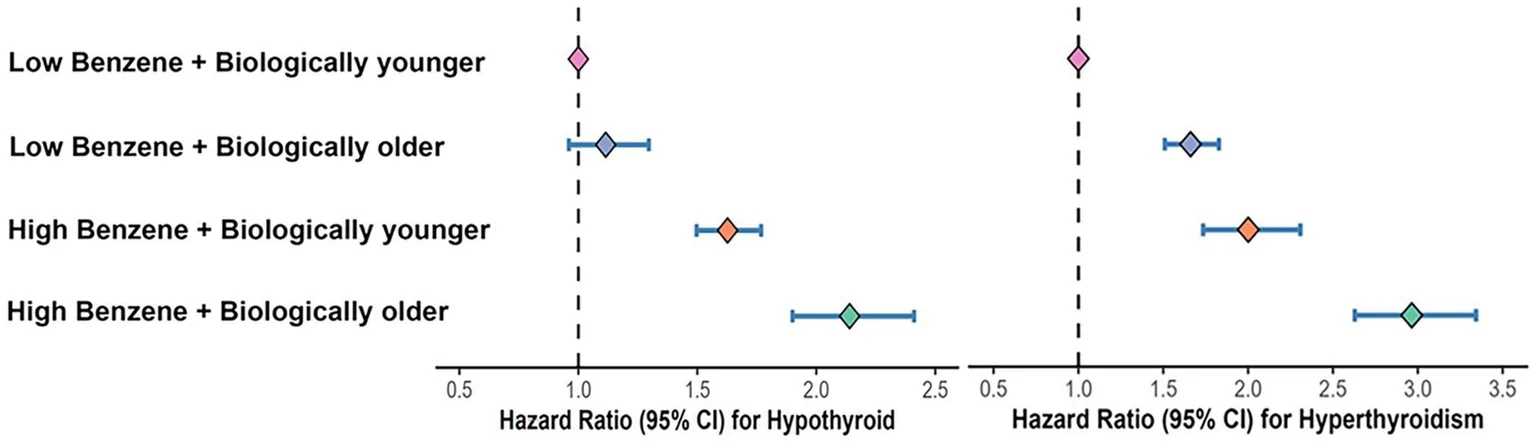
Additive interaction between biologically accelerated aging and air pollution on thyroid dysfunction. Models were adjusted for age (continuous, years), sex (male and female), ethnicity (white people and others), TDI (low, moderate, high), BMI (<30 kg/m^2^, ≥30 kg/m^2^), tobacco smoking (never, current or former smokers), alcohol consumption (never, current or former drinkers), physical activity (inactive, low, moderate, high), health diet (yes, no), and use of thyroid-related medications (yes, no), PM_2.5_. BMI, body mass index; TDI, townsend deprivation index; PM_2.5_, particulate matter with a diameter of <2.5 μm.

**Table 3:** Mediating effect of accelerated biological aging between benzene exposure and thyroid dysfunction.

Benzene exposure	Total effect	*p*-value	Natural direct effect	*p*-value	Natural indirect effect	*p*-value	Proportion mediated, %	*p*-value
Hypothyroidism								
Benzene(continuous)	0.16 (0.14, 0.19)	<0.01	0.16 (0.14, 0.19)	<0.01	0.001 (0.001, 0.003)	<0.01	0.48 (0.20, 0.76)	<0.01
Benzene(quartiles)								
Quartile 1	Ref.		Ref.		Ref.		Ref.	
Quartile 2	0.28 (0.21, 0.34)	<0.01	0.27 (0.21, 0.34)	<0.01	0.001 (0.000, 0.002)	<0.01	0.47 (0.12, 0.81)	<0.01
Quartile 3	0.32 (0.25, 0.38)	<0.01	0.32 (0.25, 0.38)	<0.01	0.003 (0.002, 0.004)	<0.01	1.07 (0.52, 1.61)	<0.01
Quartile 4	0.49 (0.41, 0.57)	<0.01	0.49 (0.41, 0.57)	<0.01	0.004 (0.002, 0.006)	<0.01	0.98 (0.52, 1.45)	<0.01
Hyperthyroidism								
Benzene (continuous)	0.19 (0.16, 0.22)	<0.01	0.19 (0.16, 0.22)	<0.01	0.002 (0.001, 0.003)	<0.01	1.17 (0.57, 1.76)	<0.01
Benzene (quartiles)								
Quartile 1	Ref.		Ref.		Ref.		Ref.	
Quartile 2	0.21 (0.13, 0.28)	<0.01	0.20 (0.12, 0.28)	<0.01	0.004 (0.002, 0.007)	<0.01	2.35 (0.88, 3.83)	<0.01
Quartile 3	0.17 (0.09, 0.25)	<0.01	0.16 (0.08, 0.24)	<0.01	0.008 (0.005, 0.011)	<0.01	5.00 (2.10, 7.80)	<0.01
Quartile 4	0.46 (0.38, 0.55)	<0.01	0.45 (0.37, 0.54)	<0.01	0.011 (0.008, 0.015)	<0.01	3.07 (2.05, 4.08)	<0.01

The models were adjusted for age (continuous, years), sex (male and female), ethnicity (white people and others), TDI (low, moderate, high), BMI (<30 kg/m^2^, ≥30 kg/m^2^), tobacco smoking (never, current or former smokers), alcohol consumption (never, current or former drinkers), physical activity (inactive, low, moderate, high), health diet (yes, no), and use of thyroid-related medications (yes, no), PM_2.5_. BMI, body mass index; TDI, townsend deprivation index; PM_2.5_, particulate matter with a diameter of <2.5 μm; Ref., reference.

### 3.5 Sensitivity analysis

Sensitivity analyses showed consistent results when using multiple imputation to handle missing covariates (Supplementary Table S4), excluding participants with less than 2 years of follow-up (Supplementary Table S5), removing those with medication use (Supplementary Table S6), and applying inverse probability weighting to adjust for covariates (Supplementary Table S7), and using an air pollution score derived from principal component analysis to replace PM_2.5_ as an alternative adjustment for co-pollutants (Supplementary Table S8). Together, these analyses indicate a robust association between environmental benzene exposure and thyroid dysfunction.

## 4 Discussion

In this large-scale prospective cohort study, we present the first evidence demonstrating that exposure to low concentrations of environmental benzene is associated with increased risks of hypothyroidism and hyperthyroidism in a dose-dependent manner. Individuals with higher benzene concentrations and advanced biological age exhibited a significantly increased risk for incident thyroid dysfunction, with evidence of additive interactions. Moreover, accelerated biological aging played a significant mediating role in the association between benzene exposure and incident thyroid dysfunction.

In recent years, a growing body of epidemiological studies has provided support for the serious public health impact of long-term benzene exposure (26, 40, 41). However, there remains a scarcity of epidemiological studies specifically investigating the association between low-concentration environmental benzene exposure and thyroid disease. The only available study reported altered thyroid hormone levels (increased thyroxine and decreased triiodothyronine) among 42 gas station workers exposed to benzene-containing solvent mixtures (27); however, the study was limited by its cross-sectional design, small sample size, high-concentration and mixed occupational exposure setting, and its inability to establish causality. Based on a large-scale prospective cohort, the present study provides the first evidence that long-term exposure to low concentrations of benzene is significantly associated with an increased risk of hyperthyroidism.

This finding is partially supported by preclinical evidence. One study examining the effects of benzene compounds on the endocrine system in rats observed elevated serum free thyroxine levels following benzene exposure (42). Furthermore, research on zebrafish embryonic development indicated that low-dose benzene exposure had the most pronounced impact on the transcriptome and related pathways of endocrine system disorders (43). A potential mechanism is that benzene, after entering the body and being metabolized into phenol in the liver, generates a large quantity of reactive oxygen species. This process induces oxidative stress and triggers inflammatory responses, which may, in turn, increase thyroid hormone release (44–46).

Notably, benzene exposure was also found to increase the risk of hypothyroidism, a phenomenon similarly observed in other studies (47). For example, an epidemiological study involving over 30,000 participants reported that exposure to perfluorooctanoic acid (PFOA) elevated the risk of both hyperthyroidism and hypothyroidism in women (47). Long-term exposure to the air pollutant PM_2.5_ has also been linked to increased risks of hyperthyroidism and hypothyroidism in adults (14). The underlying mechanisms for these observations may involve synergistic actions across multiple pathways. Firstly, as an immunotoxicant, benzene may disrupt immune tolerance, induce autoimmune attacks targeting the thyroid, directly damage thyroid follicles, and consequently impair hormone synthesis (48). Secondly, benzene has been shown to interfere with normal pituitary cell proliferation in rats and induce a systemic inflammatory state (49). Given that thyroid hormones themselves can promote oxidative phosphorylation and the generation of endogenous reactive oxygen species (50), the observed hypothyroidism following exposure might represent an adaptive protective response by the body to mitigate excessive oxidative damage. In addition, autoimmune thyroid disease exists as a spectrum, and its progression toward hyperthyroidism or hypothyroidism depends on individual genetic backgrounds and other environmental factors (51). It is worth noting that our stratified analysis revealed an increased risk of hypothyroidism in individuals over 60 years of age, while those under 60 showed an elevated risk of hyperthyroidism. The risk of hypothyroidism rises with advancing age (52), and benzene exposure further amplifies this risk. Therefore, targeted preventive measures are warranted, such as early warnings for rising benzene concentrations and optimized outdoor activity schedules for older adults.

Benzene, a common constituent of air pollutants, has previously been shown to be associated with biological aging (35, 53, 54). It exhibits a nonlinear association with accelerated biological aging, with inflammatory response and oxidative stress being potential underlying mechanisms (44). Our findings are consistent with this established association. Furthermore, individual thyroid hormone levels are known to play a significant role in the aging process (55). Our results also indicate that accelerated biological aging increases the risk of thyroid dysfunction. Based on these findings, we investigated the joint effect of benzene exposure and biological aging on thyroid dysfunction. The results showed that the risks of both hypothyroidism and hyperthyroidism increased with rising benzene concentrations and advancing biological age. Notably, a significant additive interaction was observed between accelerated biological aging and benzene exposure for hypothyroidism. In contrast, the increased risk of hyperthyroidism associated with benzene exposure was partially mediated by accelerated biological aging. These findings suggest that the role of biological aging may differ between the two thyroid phenotypes. Specifically, the risk of hypothyroidism may be more susceptible to the combined effects of exogenous benzene exposure and pre-existing multisystem physiological decline, whereas the risk of hyperthyroidism appears to be partially influenced by toxicant-driven biological aging. However, further mechanistic studies are needed to elucidate these phenotype-specific pathways. Therefore, we propose that mitigating biological aging may be an effective strategy to alleviate thyroid dysfunction induced by environmental benzene exposure.

The primary strength of this study lies in its large-scale prospective data analysis, which provides the first evidence that low-concentration environmental benzene exposure increases the risk of thyroid dysfunction. This finding addresses a gap in the literature regarding the relationship between benzene exposure and thyroid disorders. Furthermore, we revealed a significant nonlinear dose–response relationship between benzene exposure levels and the risk of thyroid dysfunction, offering stronger support for a potential causal association. In addition, we examined the additive interaction and mediating role of biological aging in the association between benzene exposure and thyroid dysfunction, offering potential pathways for the prevention of thyroid diseases. However, several limitations should be noted. First, environmental benzene exposure was estimated based on participants’ residential addresses, and individual differences in benzene absorption and metabolism may not be fully captured. Future studies could incorporate personal monitoring devices to obtain more accurate individual-level exposure data. Second, baseline benzene concentrations may not fully represent long-term exposure levels; dynamic monitoring of benzene concentrations over time is needed in future research. Third, the use of hospital inpatient records may have missed milder community-managed cases, potentially introducing selection bias if disease severity or healthcare-seeking behavior is related to exposure status. Additionally, undiagnosed subclinical thyroid dysfunction at baseline cannot be entirely ruled out despite the 2-year exclusion. Nonetheless, the positive association remained robust across sensitivity analyses. Fourth, we adjusted for confounding factors using inverse probability weighting and performed multiple imputation for missing covariates, and sensitivity analyses yielded consistent results. However, benzene often co-occurs with other traffic-related pollutants such as NO_2_ and NOx; we adjusted for PM_2.5_ as a representative indicator, and residual confounding from other co-pollutants cannot be completely excluded. Nevertheless, a sensitivity analysis using an air pollution score derived from principal component analysis in place of PM_2.5_ yielded consistent results. Finally, although we observed a statistically significant mediating effect of accelerated biological aging, the proportion mediated was modest (<5%), which limits its clinical significance and suggests that other pathways likely play more important roles.

## 5 Conclusion

Long-term benzene exposure in adults is associated with an increased risk of both hyperthyroidism and hypothyroidism, particularly among individuals with higher benzene levels and older biological age. Notably, accelerated biological aging exhibits significant additive and mediating effects on the association between benzene exposure and incident thyroid dysfunction, suggesting a potential underlying biological mechanism. These findings provide novel epidemiological evidence for the prevention of thyroid disorders and suggest that thyroid disease prevention strategies and air pollution control standards should consider the significance of environmental benzene exposure.

## Data Availability

Publicly available datasets were analyzed in this study. This data can be found at: https://biobank.ndph.ox.ac.uk/showcase/.

## Footnotes

1
https://www.ukbiobank.ac.uk
