# Supplementary material for: Long-term exposure to low concentrations of ambient benzene with incident thyroid dysfunction and the mediating role of accelerated biological aging
Source: Front Public Health. 2026 Sep 17;14:1964801. doi: 10.3389/fpubh.2026.1964801 (PMC13626889; doi:10.3389/fpubh.2026.1964801)
Supplement: Supplementary file 1 [file Table_1.docx]

**Supplementary data**

**Table S1. The definitions of diet component used in the UK biobank study**

| **Diet component** | **Intake goal** | **Data field IDs** | **Amount per serving** |
| --- | --- | --- | --- |
| Fruit | 3 servings/day | 1309 | 1 piece |
|  |  | 1319 | 5 pieces |
| Vegetable | 3 servings/day | 1289 | 3 heaped tablespoons |
|  |  | 1299 |  |
| (Shell) fish | ≥2 servings/week | 1329 | Once/week |
|  |  | 1339 |  |
| Red and processed meat | ≤2 servings/week | 1359 | Once/week |
|  |  | 1369 |  |
|  |  | 1379 |  |
|  |  | 1389 |  |
|  |  | 1349 | 1 piece/day |
| Whole grains | 3 servings/day | 1438, 1448 | 1 slice/day |
|  |  | 1458, 1468 | 1 bowl/day |
| Sugar-sweetened beverages | Don not drink | 6155 | Only 0 servings were possible here |
| Low-fat dairy | Intake | 1418 | Intake of semi-skimmed, skimmed, and soya milk was possible here |

**Table S2. HR (95% CI) for incidence based on PhenoAge acceleration**

|  | **PhenoAge acceleration (quartiles)** | | | |  |
| --- | --- | --- | --- | --- | --- |
|  | Quartile 1 | Quartile 2 | Quartile 3 | Quartile 4 | *P* for trend |
| **Hypothyroidism** | | | | | |
| Model 1 | Ref. | 1.17 (1.10, 1.26) | 1.39 (1.30, 1.49) | 1.85 (1.73, 1.97) | < 0.001 |
| Model 2 | Ref. | 1.11 (1.04, 1.19) | 1.26 (1.18, 1.35) | 1.55 (1.45, 1.66) | < 0.001 |
| Model 3 | Ref. | 1.11 (1.03, 1.18) | 1.25 (1.16, 1.33) | 1.49 (1.39, 1.59) | < 0.001 |
| **Hyperthyroidism** | | | | | |
| Model 1 | Ref. | 1.17 (1.08, 1.28) | 1.42 (1.31, 1.53) | 2.56 (2.38, 2.75) | < 0.001 |
| Model 2 | Ref. | 1.09 (1.01, 1.19) | 1.23 (1.13, 1.33) | 1.93 (1.79, 2.09) | < 0.001 |
| Model 3 | Ref. | 1.09 (1.01, 1.19) | 1.23 (1.13, 1.34) | 1.93 (1.79, 2.09) | < 0.001 |

Model 1: Age (continuous, years), sex (female, male) were adjusted.
Model 2: Age (continuous, years), sex (male and female), ethnicity (white people and others), TDI (low, moderate, high), BMI (<30 kg/m^2^, ≥30 kg/m^2^), tobacco smoking (never, current or former smokers), alcohol consumption (never, current or former drinkers), physical activity (inactive, low, moderate, high), health diet (yes, no) were adjusted.
Model 3: Age (continuous, years), sex (male and female), ethnicity (white people and others), TDI (low, moderate, high), BMI (<30 kg/m^2^, ≥30 kg/m^2^), tobacco smoking (never, current or former smokers), alcohol consumption (never, current or former drinkers), physical activity (inactive, low, moderate, high), health diet (yes, no) , and use of thyroid-related medications (yes, no), PM_2.5_ were adjusted.

*P* for trend was tested by using ordered integer values.

**Table S3. RERI, AP for cumulative interactions between benzene and accelerated biological aging**

| **Benzene** | **PhenoAge acceleration** | |
| --- | --- | --- |
|  | **RERI** （**95 % CI**） | **AP** （**95 % CI**） |
| **Hypothyroidism** | | |
| Quartile 2 | 0.21 (-0.04, 0.46) | 0.13 (-0.02, 0.27) |
| Quartile 3 | 0.43 (0.18, 0.68) | 0.23 (0.11, 0.35) |
| Quartile 4 | 0.40 (0.13, 0.66) | 0.19 (0.07, 0.30) |
| **Hyperthyroidism** | | |
| Quartile 2 | 0.21 (-0.15, 0.56) | 0.09 (-0.06, 0.23) |
| Quartile 3 | 0.12 (-0.22, 0.46) | 0.05 (-0.10, 0.20) |
| Quartile 4 | 0.30 (-0.06, 0.67) | 0.10 (-0.02, 0.22) |

**Table S4. Association between benzene exposure and thyroid dysfunction after multiple imputation of covariates**

|  | **Benzene(continuous)** | **Benzene(quartiles)** | | | |  |
| --- | --- | --- | --- | --- | --- | --- |
|  | HR (95% CI) for a 1 μg/m^3^ increase | Quartile 1 | Quartile 2 | Quartile 3 | Quartile 4 | P for trend |
| **Hypothyroidism** | | | | | | |
| Model 1 | 1.29 (1.27, 1.32) | Ref. | 1.71 (1.61, 1.82) | 1.87 (1.75, 1.98) | 2.22 (2.09, 2.36) | <0.001 |
| Model 2 | 1.30 (1.28, 1.33) | Ref. | 1.71 (1.61, 1.82) | 1.84 (1.73, 1.96) | 2.16 (2.03, 2.30) | <0.001 |
| Model 3 | 1.31 (1.28, 1.34) | Ref. | 1.73 (1.62, 1.84) | 1.86 (1.75, 1.98) | 2.18 (2.04, 2.33) | <0.001 |
| **Hyperthyroidism** | | | | | | |
| Model 1 | 1.51 (1.48, 1.54) | Ref. | 1.70 (1.58, 1.82) | 1.83 (1.70, 1.96) | 3.02 (2.82, 3.24) | <0.001 |
| Model 2 | 1.36 (1.33, 1.39) | Ref. | 1.72 (1.60, 1.85) | 1.75 (1.63, 1.88) | 2.36 (2.20, 2.54) | <0.001 |
| Model 3 | 1.35 (1.32, 1.38) | Ref. | 1.69 (1.57, 1.82) | 1.70 (1.58, 1.83) | 2.26 (2.10, 2.44) | <0.001 |

The models were adjusted for age (continuous, years), sex (male and female), ethnicity (white people and others), TDI (low, moderate, high), BMI (<30 kg/m^2^, ≥30 kg/m^2^), tobacco smoking (never, current or former smokers), alcohol consumption (never, current or former drinkers), physical activity (inactive, low, moderate, high), health diet (yes, no) , and use of thyroid-related medications (yes, no), PM_2.5_. BMI: body mass index; TDI, Townsend deprivation index; PM_2.5_, particulate matter with diameter < 2.5 μm. *P* for trend was tested by using ordered integer values.

**Table S5. Association of benzene exposure with thyroid dysfunction after excluding participants with less than 2 years of follow-up**

|  | **Benzene(continuous)** | **Benzene(quartiles)** | | | |  |
| --- | --- | --- | --- | --- | --- | --- |
|  | HR (95% CI) for 1 μg/m^3^ increase | Quartile 1 | Quartile 2 | Quartile 3 | Quartile 4 | P for trend |
| **Hypothyroidism** | | | | | | |
| Model 1 | 1.17 (1.15, 1.19) | Ref. | 1.28 (1.20 1.36) | 1.34 (1.27, 1.43) | 1.57 (1.48, 1.67) | <0.001 |
| Model 2 | 1.16 (1.13, 1.18) | Ref. | 1.27 (1.19, 1.35) | 1.32 (1.24, 1.40) | 1.49 (1.40, 1.59) | <0.001 |
| Model 3 | 1.16 (1.13, 1.19) | Ref. | 1.26 (1.19, 1.34) | 1.31 (1.23, 1.39) | 1.49 (1.40, 1.59) | <0.001 |
| **Hyperthyroidism** | | | | | | |
| Model 1 | 1.38 (1.35, 1.41) | Ref. | 1.29 (1.20, 1.38) | 1.32 (1.23, 1.42) | 2.20 (2.06, 2.35) | <0.001 |
| Model 2 | 1.21 (1.18, 1.24) | Ref. | 1.25 (1.16, 1.34) | 1.21 (1.12, 1.30) | 1.62 (1.51, 1.73) | <0.001 |
| Model 3 | 1.20 (1.17, 1.23) | Ref. | 1.23 (1.15, 1.33) | 1.18 (1.10, 1.27) | 1.56 (1.45, 1.68) | <0.001 |

The models were adjusted for age (continuous, years), sex (male and female), ethnicity (white people and others), TDI (low, moderate, high), BMI (<30 kg/m^2^, ≥30 kg/m^2^), tobacco smoking (never, current or former smokers), alcohol consumption (never, current or former drinkers), physical activity (inactive, low, moderate, high), health diet (yes, no) , and use of thyroid-related medications (yes, no), PM_2.5_. BMI: body mass index; TDI, Townsend deprivation index; PM_2.5_, particulate matter with diameter < 2.5 μm. *P* for trend was tested by using ordered integer values.

**Table S6. Association of benzene exposure with thyroid dysfunction after excluding participants with medication usage**

|  | **Benzene(continuous)** | **Benzene(quartiles)** | | | |  |
| --- | --- | --- | --- | --- | --- | --- |
|  | HR (95% CI) for a 1 μg/m^3^ increase | Quartile 1 | Quartile 2 | Quartile 3 | Quartile 4 | P for trend |
| **Hypothyroidism** | | | | | | |
| Model 1 | 1.17 (1.15, 1.19) | Ref. | 1.30 (1.22, 1.38) | 1.36 (1.28, 1.44) | 1.59 (1.50, 1.70) | <0.001 |
| Model 2 | 1.16 (1.13, 1.18) | Ref. | 1.29 (1.21, 1.37) | 1.33 (1.25, 1.41) | 1.51 (1.42, 1.61) | <0.001 |
| Model 3 | 1.16 (1.14, 1.19) | Ref. | 1.29 (1.21, 1.38) | 1.33 (1.25, 1.42) | 1.52 (1.42, 1.63) | <0.001 |
| **Hyperthyroidism** | | | | | | |
| Model 1 | 1.40 (1.37, 1.43) | Ref. | 1.30 (1.21, 1.40) | 1.35 (1.26, 1.45) | 2.28 (2.14, 2.44) | <0.001 |
| Model 2 | 1.23 (1.20, 1.26) | Ref. | 1.27 (1.18, 1.36) | 1.23 (1.15, 1.32) | 1.67 (1.56, 1.80) | <0.001 |
| Model 3 | 1.22 (1.19, 1.25) | Ref. | 1.25 (1.17, 1.35) | 1.21 (1.12, 1.30) | 1.62 (1.51, 1.74) | <0.001 |

The models were adjusted for age (continuous, years), sex (male and female), ethnicity (white people and others), TDI (low, moderate, high), BMI (<30 kg/m^2^, ≥30 kg/m^2^), tobacco smoking (never, current or former smokers), alcohol consumption (never, current or former drinkers), physical activity (inactive, low, moderate, high), health diet (yes, no), PM_2.5_. BMI: body mass index; TDI, Townsend deprivation index; PM_2.5_, particulate matter with diameter < 2.5 μm. *P* for trend was tested by using ordered integer values.

**Table S7. Association between benzene exposure and thyroid dysfunction based on inverse probability weighted analysis**

|  | **Benzene(quartiles)** | | | |  |
| --- | --- | --- | --- | --- | --- |
|  | Quartile 1 | Quartile 2 | Quartile 3 | Quartile 4 | P for trend |
| **Hypothyroidism** | | | | | |
| Model 1 | Ref. | 1.23 (1.08, 1.40) | 1.18 (1.04, 1.34) | 1.54 (1.36, 1.74) | <0.001 |
| Model 2 | Ref. | 1.22 (1.08, 1.39) | 1.18 (1.03, 1.34) | 1.54 (1.36, 1.74) | <0.001 |
| Model 3 | Ref. | 1.23 (1.08, 1.39) | 1.19 (1.05, 1.34) | 1.55 (1.38, 1.74) | <0.001 |
| **Hyperthyroidism** | | | | | |
| Model 1 | Ref. | 1.19 (1.05, 1.35) | 1.16 (1.02, 1.32) | 1.59 (1.41, 1.80) | <0.001 |
| Model 2 | Ref. | 1.19 (1.04, 1.35) | 1.16 (1.02, 1.31) | 1.59 (1.40, 1.80) | <0.001 |
| Model 3 | Ref. | 1.19 (1.05, 1.35) | 1.16 (1.03, 1.31) | 1.60 (1.42, 1.80) | <0.001 |

The models were adjusted for age (continuous, years), sex (male and female), ethnicity (white people and others), TDI (low, moderate, high), BMI (<30 kg/m^2^, ≥30 kg/m^2^), tobacco smoking (never, current or former smokers), alcohol consumption (never, current or former drinkers), physical activity (inactive, low, moderate, high), health diet (yes, no) , and use of thyroid-related medications (yes, no), PM_2.5_. BMI: body mass index; TDI, Townsend deprivation index; PM_2.5_, particulate matter with diameter < 2.5 μm. *P* for trend was tested by using ordered integer values.

**Table S8. Association of benzene exposure with thyroid dysfunction after adjusting for an air pollution score derived from principal component analysis**

|  | **Benzene(continuous)** | **Benzene(quartiles)** | | | |  |
| --- | --- | --- | --- | --- | --- | --- |
|  | HR (95% CI) for a 1 μg/m^3^ increase | Quartile 1 | Quartile 2 | Quartile 3 | Quartile 4 | P for trend |
| **Hypothyroidism** | | | | | | |
| Model 1 | 1.19 (1.17, 1.22) | Ref. | 1.31 (1.23, 1.39) | 1.40 (1.32, 1.49) | 1.65 (1.56, 1.76) | <0.001 |
| Model 2 | 1.18 (1.15, 1.21) | Ref. | 1.30 (1.22, 1.38) | 1.37 (1.29, 1.46) | 1.57 (1.47, 1.68) | <0.001 |
| Model 3 | 1.17 (1.15, 1.20) | Ref. | 1.29 (1.21, 1.37) | 1.36 (1.28, 1.45) | 1.55 (1.45, 1.66) | <0.001 |
| **Hyperthyroidism** | | | | | | |
| Model 1 | 1.39 (1.36, 1.42) | Ref. | 1.32 (1.22, 1.42) | 1.36 (1.26, 1.46) | 2.27 (2.11, 2.43) | <0.001 |
| Model 2 | 1.23 (1.20, 1.26) | Ref. | 1.28 (1.19, 1.38) | 1.24 (1.15, 1.33) | 1.66 (1.54, 1.79) | <0.001 |
| Model 3 | 1.23 (1.20, 1.26) | Ref. | 1.28 (1.19, 1.37) | 1.24 (1.15, 1.33) | 1.66 (1.54, 1.79) | <0.001 |

The models were adjusted for age (continuous, years), sex (male and female), ethnicity (white people and others), TDI (low, moderate, high), BMI (<30 kg/m^2^, ≥30 kg/m^2^), tobacco smoking (never, current or former smokers), alcohol consumption (never, current or former drinkers), physical activity (inactive, low, moderate, high), health diet (yes, no), air pollution score. BMI: body mass index; TDI, Townsend deprivation index. *P* for trend was tested by using ordered integer values.

Fig. S1 Flow chart of participants included in the study.

Fig. S2 Directed acyclic graph (DAG) for covariate selection.

Fig. S3 Subgroup Analysis of the Association Between Environmental Benzene Exposure and Thyroid Dysfunction.

Fig. S4 Exposure-response curves of accelerated biological aging and thyroid dysfunction. Models were adjusted for age (continuous, years), sex (male and female), ethnicity (white people and others), TDI (low, moderate, high), BMI (<30 kg/m^2^, ≥30 kg/m^2^), tobacco smoking (never, current or former smokers), alcohol consumption (never, current or former drinkers), physical activity (inactive, low, moderate, high), health diet (yes, no) , and use of thyroid-related medications (yes, no), PM_2.5_. BMI: body mass index; TDI, Townsend deprivation index; PM_2.5_, particulate matter with diameter < 2.5 μm.

Fig. S5 Exposure-response curves of environmental benzene exposure and accelerated biological aging. Models were adjusted for age (continuous, years), sex (male and female), ethnicity (white people and others), TDI (low, moderate, high), BMI (< 30 kg/m^2^, ≥ 30 kg/m^2^), tobacco smoking (never, current or former smokers), alcohol consumption (never, current or former drinkers), physical activity (inactive, low, moderate, high), health diet (yes, no) , and use of thyroid-related medications (yes, no), PM_2.5_. BMI: body mass index; TDI, Townsend deprivation index; PM_2.5_, particulate matter with diameter < 2.5 μm.
